# Environmental effects of stratospheric ozone depletion, UV radiation, and interactions with climate change: UNEP Environmental Effects Assessment Panel, Update 2021

**DOI:** 10.1007/s43630-022-00176-5

**Published:** 2022-02-21

**Authors:** P. W. Barnes, T. M. Robson, P. J. Neale, C. E. Williamson, R. G. Zepp, S. Madronich, S. R. Wilson, A. L. Andrady, A. M. Heikkilä, G. H. Bernhard, A. F. Bais, R. E. Neale, J. F. Bornman, M. A. K. Jansen, A. R. Klekociuk, J. Martinez-Abaigar, S. A. Robinson, Q.-W. Wang, A. T. Banaszak, D.-P. Häder, S. Hylander, K. C. Rose, S.-Å. Wängberg, B. Foereid, W.-C. Hou, R. Ossola, N. D. Paul, J. E. Ukpebor, M. P. S. Andersen, J. Longstreth, T. Schikowski, K. R. Solomon, B. Sulzberger, L. S. Bruckman, K. K. Pandey, C. C. White, L. Zhu, M. Zhu, P. J. Aucamp, J. B. Liley, R. L. McKenzie, M. Berwick, S. N. Byrne, L. M. Hollestein, R. M. Lucas, C. M. Olsen, L. E. Rhodes, S. Yazar, A. R. Young

**Affiliations:** 1 Biological Sciences and Environment Program, Loyola University New Orleans, New Orleans, USA; 2 Organismal and Evolutionary Biology (OEB), Viikki Plant Science Centre (ViPS), University of Helsinki, Helsinki, Finland; 3 Smithsonian Environmental Research Center, Edgewater, USA; 4 Department of Biology, Miami University, Oxford, USA; 5 ORD/CEMM, US Environmental Protection Agency, Athens, GA USA; 6 Atmospheric Chemistry Observations and Modeling Laboratory, National Center for Atmospheric Research, Boulder, USA; 7 School of Earth, Atmospheric and Life Sciences, University of Wollongong, Wollongong, Australia; 8 Chemical and Biomolecular Engineering, North Carolina State University, Apex, USA; 9 Finnish Meteorological Institute, Helsinki, Finland; 10 Biospherical Instruments Inc, San Diego, USA; 11 Laboratory of Atmospheric Physics, Department of Physics, Aristotle University, Thessaloniki, Greece; 12 Population Health Department, QIMR Berghofer Medical Research Institute, Brisbane, Australia; 13 Food Futures Institute, Murdoch University, Perth, Australia; 14 BEES, University College Cork, Cork, Ireland; 15 Antarctic Climate Program, Australian Antarctic Division, Kingston, Australia; 16 Faculty of Science and Technology, University of La Rioja, La Rioja, Logroño, Spain; 17 Securing Antarctica’s Environmental Future, Global Challenges Program and School of Earth, Atmospheric and Life Sciences, University of Wollongong, Wollongong, Australia; 18 Institute of Applied Ecology, Chinese Academy of Sciences (CAS), Shenyang, China; 19 Unidad Académica De Sistemas Arrecifales, Universidad Nacional Autónoma De México, Puerto Morelos, Mexico; 20 Department of Biology, Friedrich-Alexander University, Möhrendorf, Germany; 21 Centre for Ecology and Evolution in Microbial Model Systems-EEMiS, Linnaeus University, Kalmar, Sweden; 22 Biological Sciences, Rensselaer Polytechnic Institute, Troy, USA; 23 Marine Sciences, University of Gothenburg, Gothenburg, Sweden; 24 Environment and Natural Resources, Norwegian Institute of Bioeconomy Research, Ås, Norway; 25 Environmental Engineering, National Cheng Kung University, Tainan, Taiwan; 26 Environmental System Science (D-USYS), ETH Zürich, Zürich, Switzerland; 27 Lancaster Environment Centre, Lancaster University, Lancaster, UK; 28 Chemistry Department, Faculty of Physical Sciences, University of Benin, Benin City, Nigeria; 29 Department of Chemistry and Biochemistry, California State University, Northridge, USA; 30 Department of Chemistry, University of Copenhagen, Copenhagen, Denmark; 31 The Institute for Global Risk Research, LLC, Bethesda, USA; 32 Research Group of Environmental Epidemiology, Leibniz Institute of Environmental Medicine, Düsseldorf, Germany; 33 Centre for Toxicology, School of Environmental Sciences, University of Guelph, Guelph, Canada; 34 Academic Guest, Swiss Federal Institute of Aquatic Science and Technology, 8600 Dübendorf, Switzerland; 35 Materials Science and Engineering, Case Western Reserve University, Cleveland, USA; 36 Wood Processing Division, Institute of Wood Science and Technology, Bangalore, India; 37 Polymer Science and Materials Chemistry (PSMC), Exponent, Bethesda USA; 38 College of Materials Science and Engineering, Donghua University, Shanghai, China; 39 State Key Laboratory for Modification of Chemical Fibers and Polymer Materials, Donghua University, Shanghai, China; 40 Ptersa Environmental Consultants, Pretoria, South Africa; 41 National Institute of Water and Atmospheric Research, Alexandra, New Zealand; 42 Internal Medicine, University of New Mexico, Albuquerque, USA; 43 Applied Medical Science, University of Sydney, Sydney, Australia; 44 Department of Dermatology, Erasmus MC Cancer Institute, Rotterdam, The Netherlands; 45 National Centre for Epidemiology and Population Health, Australian National University, Canberra, Australia; 46 Photobiology Unit, Dermatology Research Centre, School of Biological Sciences, Faculty of Biology Medicine and Health, University of Manchester, Manchester, UK; 47 Garvan Institute of Medical Research, Sydney, Australia; 48 St John’s Institute of Dermatology, King’s College London (KCL), London, UK

## Abstract

The Environmental Effects Assessment Panel of the Montreal Protocol under the United Nations Environment Programme evaluates effects on the environment and human health that arise from changes in the stratospheric ozone layer and concomitant variations in ultraviolet (UV) radiation at the Earth’s surface. The current update is based on scientific advances that have accumulated since our last assessment (Photochem and Photobiol Sci 20(1):1–67, 2021). We also discuss how climate change affects stratospheric ozone depletion and ultraviolet radiation, and how stratospheric ozone depletion affects climate change. The resulting interlinking effects of stratospheric ozone depletion, UV radiation, and climate change are assessed in terms of air quality, carbon sinks, ecosystems, human health, and natural and synthetic materials. We further highlight potential impacts on the biosphere from extreme climate events that are occurring with increasing frequency as a consequence of climate change. These and other interactive effects are examined with respect to the benefits that the Montreal Protocol and its Amendments are providing to life on Earth by controlling the production of various substances that contribute to both stratospheric ozone depletion and climate change.

## Stratospheric ozone, UV radiation, and climate interactions

Since the last 2020 EEAP Update Assessment [1], new information on the beneficial effects of the Montreal Protocol on the stratospheric ozone layer has become available and is assessed. Other focus areas of this section are: implications of the contrast between the unusually weak Antarctic vortex in 2019 and the large and long-lasting 2020 Antarctic ozone hole, the effects on weather at northern mid-latitudes resulting from the 2020 Arctic low-ozone episode, and recent projections of Arctic ozone and UV radiation linked to climate change.

### The weak Antarctic vortex in 2019 favoured extreme weather and wildfires in Australia, but the atmospheric conditions that contributed to this event appear less likely under future climate scenarios

The unusual warming of the Antarctic stratosphere in September 2019 favoured the extremely dry conditions observed during the summer of 2019/20 in the Southern Hemisphere [2] that contributed to the devastating “2019/2020 Black Summer'' wildfires in Australia [3]. Additional studies [4–6] have further reinforced the link between the unusually weak Antarctic vortex in 2019 and the ensuing dry weather in the Southern Hemisphere. However, stratospheric warming events, such as that observed in 2019, are less likely in a future climate [6] as increasing concentrations of greenhouse gases (GHG) will cool the stratosphere. Furthermore, modelling studies suggest that summertime precipitation in the Southern Hemisphere, with some regions projected to get drier and others wetter, will be more affected by future increases in the concentration of GHGs and warming of the tropical upper troposphere than by stratospheric ozone recovery resulting from the implementation of the Montreal Protocol [7].

### The 2020 Antarctic ozone hole led to record-breaking increases in UV-B radiation but this does not imply that the Montreal Protocol is not effective in reducing polar ozone depletion

**Fig. 1:**
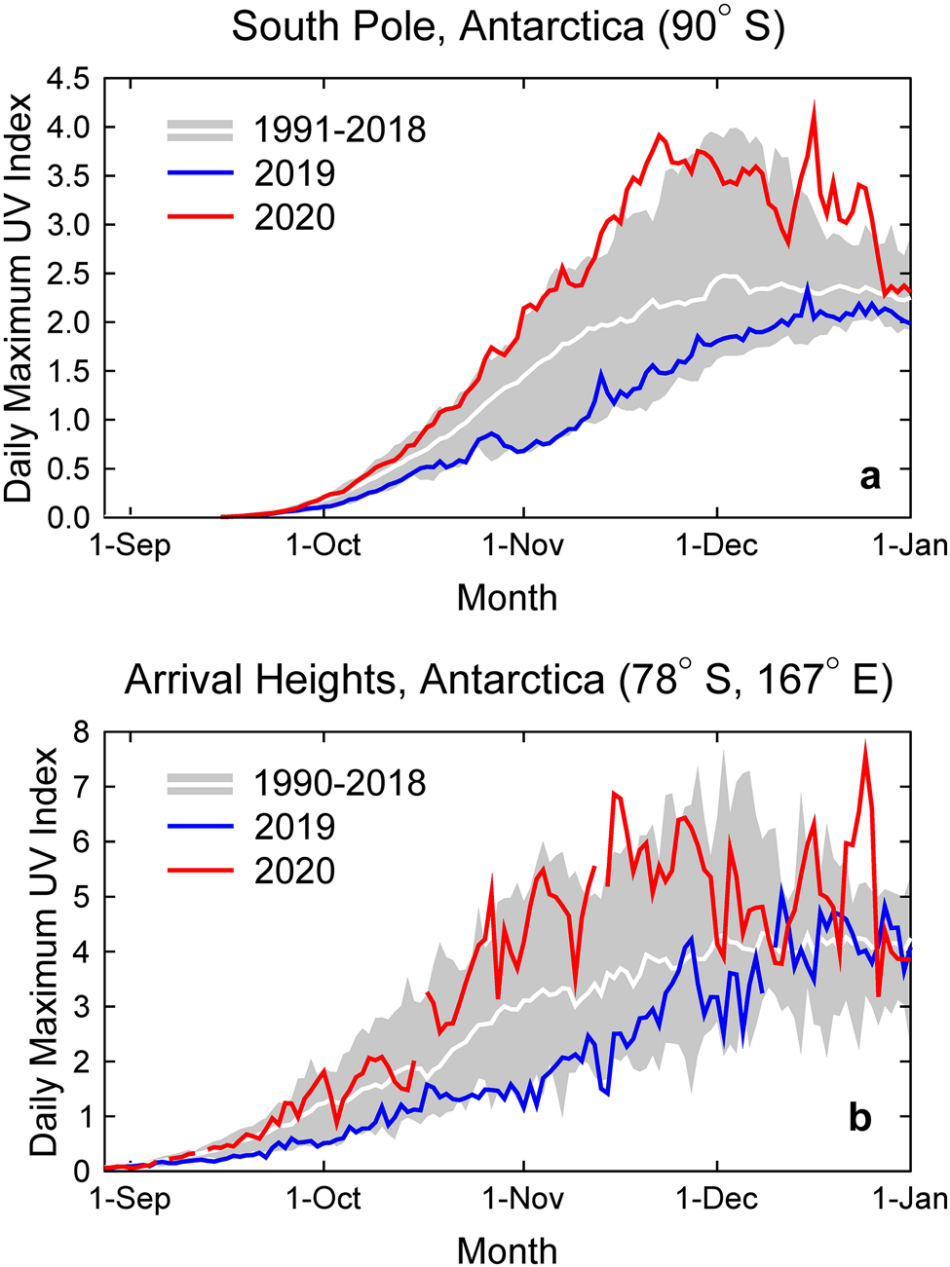
Daily maximum UV index (UVI) measured at the South Pole (**a**) and Arrival Heights (**b**) in 2019 (blue line) and 2020 (red line), compared with the average (white line) and the range (grey shading) of daily maximum observations of the years indicated in the legends. The UVI was calculated from spectra measured by SUV-100 spectroradiometers. Up to 2009, the instruments were part of the NSF UV monitoring network [13] and they are now a node in the NOAA Antarctic UV Monitoring Network (https://www.esrl.noaa.gov/gmd/grad/antuv/). Consistent data processing methods were applied for all years [14, 15]. In 2020, the UVI was typically above the long-term average at both sites due to the sustained and deep stratospheric ozone hole in that year. Conversely, the UVI in 2019 was close to the lower limit of historical observations because warming of the Antarctic stratosphere produced one of the smallest ozone holes on record in that year [1, 8]

In contrast to the spring of 2019, when the Antarctic stratosphere was warm, the lack of planetary waves (i.e. large-scale perturbations in atmospheric circulation) during the 2020 austral spring resulted in a cold and stable stratospheric vortex over Antarctica, which created conditions favourable for persistent ozone depletion [8]. Additionally, ozone loss in early spring enhanced the strength and persistence of the vortex later in the year [9]. These conditions led to the longest-lived Antarctic ozone hole in the observational record. Consequently, record-low total ozone columns in November and December 2020 resulted in unusually high levels of UV-B radiation (280–315 nm) in the Antarctic region [8, 10]. At Arrival Heights (78° S), the UV index (UVI) reached a new all-time site record of 7.8 on 23 December (Fig. 1), exceeding the previous record for this day by nearly 50% [8]. At Marambio (64° S), a station located near the Antarctic Peninsula, the daily maximum UVI exceeded 12 on several days in late November and early December 2020 [8], coinciding with the start of the Antarctic growing season and potentially causing photodamage to plants and animals. The UVI values measured at Marambio were amongst the highest recorded during the last 30 years in Antarctica and are comparable with summertime UVI maxima at subtropical sites such as San Diego (32° N) [11].

Despite the abnormally low stratospheric ozone and high UVI in late spring of 2020, the healing of the Antarctic ozone hole due to the implementation of the Montreal Protocol is still on track, as evidenced by the observed continuing decline in stratospheric ozone depletion during September—the key month for chemical ozone destruction [8]—and the general trend of all metrics quantifying Antarctic ozone depletion pointing towards recovery of the ozone hole [12].

### Unprecedented Arctic ozone depletion in 2020 contributed to abnormally high springtime temperatures across Asia and Europe

Years with a strong Arctic polar vortex and associated significant ozone depletion have been linked to widespread climate anomalies across the Northern Hemisphere [16]. As predicted by this study, the exceptionally large ozone depletion that occurred in March–April 2020 [1, 17, 18] affected springtime weather in the Northern Hemisphere. Specifically, it helped to keep the Arctic Oscillation (AO^1^) in a record-high positive state through April [17], thus contributing to abnormally high temperatures across Asia and Europe [19]. Furthermore, loss of ozone modified the stability of the upper troposphere in the Siberian sector of the Arctic, leading to more high-level clouds that enhance downwelling longwave (thermal) radiation [20]. The associated anomalous surface warming in April 2020 was further amplified by the reduction in surface albedo caused by melting of snow and sea ice. For example, monthly air temperature anomalies of + 6 °C were observed over Siberia from January through May 2020 [21]. Both effects (increasing high clouds and decreasing surface albedo) would result in less UV radiation at the surface. The unprecedented depletion of Arctic ozone in the spring of 2020 contrasts with the 2020/2021 boreal winter, when a major stratospheric warming in January [22, 23] limited overall ozone loss. Such large year-to-year variations in Arctic ozone depletion, which are driven by differences in meteorological conditions, are expected to continue for as long as concentrations of ozone-depleting substances (ODSs) remain elevated [24].

### Years with large Arctic ozone loss and concomitant increases in UV radiation will continue to occur throughout the twenty-first century despite measures resulting from the Montreal Protocol

A recent study provides evidence, based on observations and modelling, that the stratosphere is getting colder during cold Arctic winters [25]. In the following spring, these colder stratospheric temperatures lead to more rapid chemical loss of ozone via catalytic processes on polar stratospheric clouds [26]. As a consequence, large ozone-depletion events like the one observed in 2020 [17] will likely recur throughout the twenty-first century despite decreasing concentrations of ODSs over this period. The magnitude of stratospheric cooling in the future will critically depend on the development of GHG concentrations and variability in the amount of water vapour in the stratosphere. Hence, anthropogenic climate change [27] has the potential to partially counteract the positive effects of the Montreal Protocol on the Arctic ozone layer.

The greatest effect on stratospheric ozone and UV radiation is projected for the “regional rivalry” SSP3-7.0 and the “fossil-fuel intensive” SSP5-8.5 “Shared Socio-economic Pathways” (or SSP^2^) for concentrations of GHGs [27, 28]. This response would be even greater if concentrations of stratospheric water vapour continue to rise during the twenty-first century as a result of anthropogenically driven increases in atmospheric concentrations of methane (CH_4_), which is oxidised in the stratosphere to water vapour [29]. Under these worst-case GHG concentration scenarios, springtime increases in UV radiation in the Arctic could be somewhat larger at the end of the twenty-first century than those observed in 2020 [30].

### Measures to reduce air pollutants in Mexico City increased the UV index by 25% between 2000 and 2019

The UVI measured on cloud-free days in Mexico City was reduced by ~ 40% in 2000 and ~ 25% in 2019 relative to the UVI expected for a clear atmosphere [31]. The increase in the UVI by ~ 25% over the intervening two decades was attributed to reductions in pollutants, i.e. in order of importance: aerosols, tropospheric ozone, NO_2_, and SO_2_. The effects of these measures may provide a scenario for other regions currently affected by photochemical smog.

Human health benefits resulting from the decrease in air pollution [32] outweigh risks—such as the potential increase in skin cancer incidence—stemming from the gradual return of UV radiation intensities to more natural levels prevailing at unpolluted areas.^3^ The effects of air quality measures implemented in Mexico City may help to project changes in UV radiation for regions that are currently still affected by heavy smog, such as South and East Asia [33, 34]. The Mexico City study also confirmed earlier findings (e.g. [35]) that the UVI at the surface of heavily polluted areas cannot be reliably estimated from satellite observations, emphasising the importance of ground-based measurements.

## Air quality

Solar UV radiation drives chemical transformations in the troposphere that have beneficial and harmful consequences. Photochemical smog, formed when urban pollutant gases (mainly nitrogen oxides (NO*x* = NO + NO_2_) and volatile organic compounds (VOCs)) are exposed to UV radiation, causes an estimated several million premature deaths per year globally. Conversely, UV radiation improves air quality by generating hydroxyl radicals (OH), which are the “cleaning agents” of the atmosphere; they remove many anthropogenic and natural gases of relevance to stratospheric ozone, climate, and air quality on all geographic scales, including molecular hydrogen (H_2_), carbon monoxide (CO), methane (CH_4_), VOCs (natural and anthropogenic), nitrogen oxides (NOx), sulphur dioxide (SO_2_), and halogenated organics such as HFCs, HCFCs, HFEs, HFOs,^4^ and methyl halides. The reaction of OH radicals with these ODS replacement compounds (HFCs, HCFCs, HFEs and HFOs) produces trifluoroacetic acid (TFA) in the atmosphere, which is transported in precipitation to the Earth’s surface. No chemical reactions that break down TFA have been identified in the environment, so although it is not highly toxic, its accumulation over time should be monitored.

### New model estimates indicate that increases in UV radiation have contributed to an increase in the tropospheric concentration of the hydroxyl radical from 1980 to 2010

**Fig. 2:**
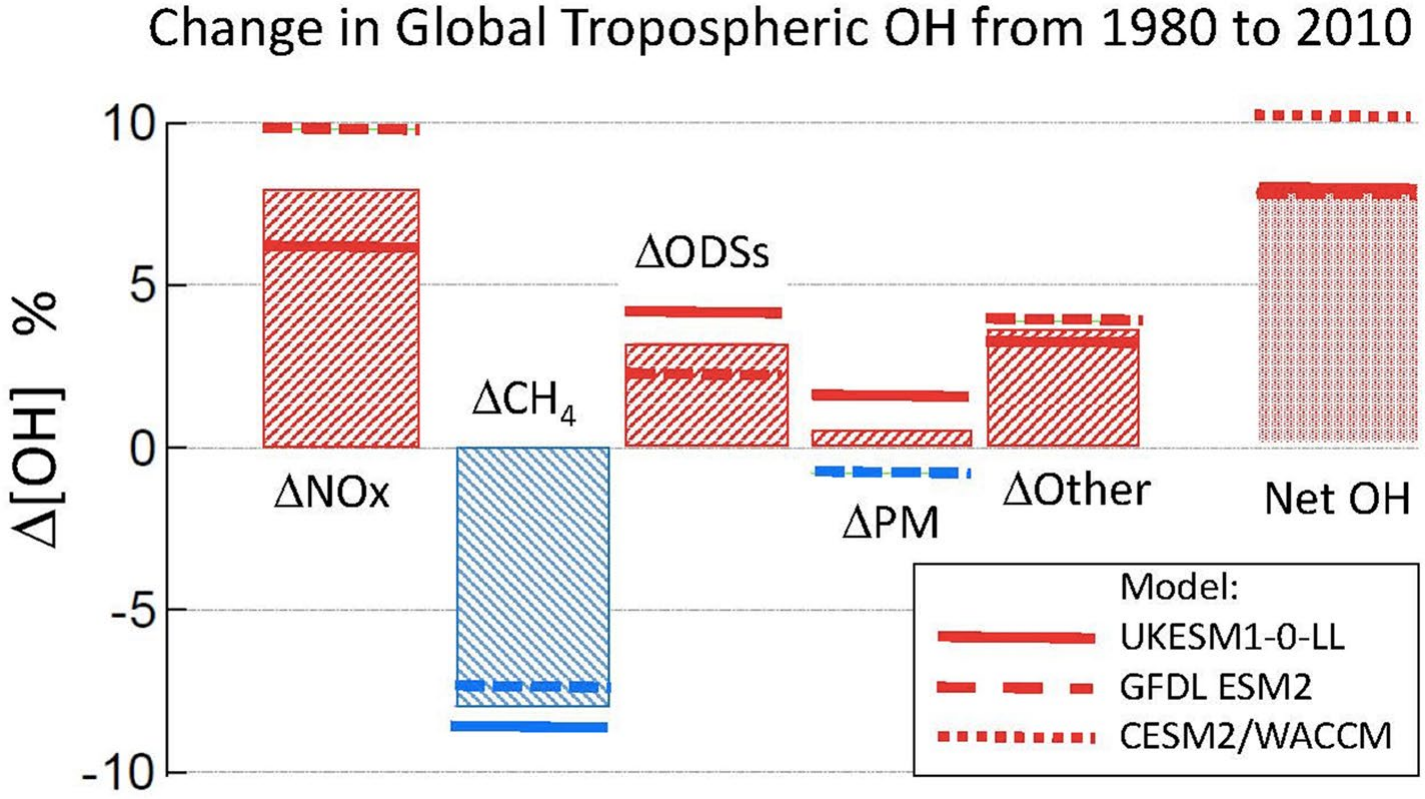
Change (%) in global tropospheric OH concentrations from 1980 to 2010, estimated with different Earth System Models (ESMs). The net change in OH (rightmost column) had contributions from increased emissions of nitrogen oxides and other precursors of tropospheric ozone (∆NO_*x*_); increased emissions of methane (∆CH_4_); accumulation of ozone-depleting substances now regulated under the Montreal Protocol (∆ODSs); emissions of particulate matter and its precursors (∆PM); and other undifferentiated changes attributed to underlying climate change as well as interactions among these separate factors (∆Other). The ODSs have contributed to the net increase in OH concentrations by depleting stratospheric ozone, thus allowing more UV radiation to penetrate into the troposphere and increase the rates of photochemical reactions that generate OH. Modified from Stevenson et al. [36]

Past trends in the tropospheric concentration of the OH radical have been estimated with models and inferred from observations, although future changes in OH are uncertain. The combined output of three computer models indicated that, from 1850 to 1980, the global concentration of OH remained constant. From 1980 to 2010 there was a net increase in OH of ~ 9% according to the models (Fig. 2). Precursors of ozone, as well as some climate change-related factors (e.g. rise in temperature and humidity), were responsible for raising tropospheric OH concentration by 8% and 4%, respectively, during this period. ODS, by increasing UV radiation, contributed about 3% to this trend. Increases in ODS occurred during this period because the Montreal Protocol had not yet been fully implemented.

Increased atmospheric CH_4_ was the main factor counteracting the trend of rising OH (by − 8%) over this period [36]. In contrast, estimates of concentrations of OH based on observed changes in the amounts of chemicals primarily removed from the atmosphere by OH, have generally indicated a decrease since around 2000 [37]. The inter-annual variability in OH was large, so the difference between modelled and measured concentration trends was not statistically significant. In particular, the increase in concentrations of atmospheric CH_4_ after 2006 appears not to be caused by a decrease in the concentration of OH, as had been previously postulated, but is more likely to have been driven by increasing emissions of CH_4_ [38].

Climate change may enhance emissions of CH_**4**_ and CO (which remove OH) by natural sources. For example, as a result of extreme wildfires in southeast Australia during 2019/2020, the annual mean burden of CO exceeded values of previous years (2001–2018) by 4- to 59-fold for this region [39]. Wildfires also enhance precursors of OH, particularly nitrous acid (HNO_2_) [40]. In the future, UV radiation and climate change will continue to interact and affect the concentration of tropospheric OH in complex ways.

### Lockdowns in response to COVID-19 caused significant short-term reductions in emissions of pollutants with both regional and global impacts

Between February and June 2020, lockdowns to mitigate spread of COVID-19 caused emissions of anthropogenic NOx to decrease globally by up to 15%, concomitant with a decrease in the total global tropospheric burden of ozone by 0.6 Tg in February 2020 and 6.5 Tg in June 2020 [41]. Improved air quality not only reduced emissions of NOx, but also the amount of tropospheric aerosols. A consequence was an increase in surface solar radiation regionally and, thus, an increased rate of UV-induced photolysis of ozone and NO_2_ [42, 43], which enhanced production of OH.

### Atmospheric concentrations of third-generation CFC-replacement compounds, HFOs and HCFOs, continue to increase, but the impact on local air quality is not significant

Uses of third-generation CFC-replacement compounds—hydrofluoroolefins (HFOs) and hydrochlorofluoroolefins (HCFOs)—are increasing in commercial applications. Ambient concentrations of HFO-1234yf and HCFO-1233zd(E) have been measured in central Europe at urban, semi-urban, and remote sites from 2011 onwards [44, 45]. In general, the concentrations of HFOs increased by a factor of 10 from 2011 to 2019, but the concentrations are low (0.7 parts per trillion (ppt) for HFO-1234yf in 2019, [45]) in comparison with the CFCs and HFCs they replace; e.g. values for CFC-11 and HFC-134a were 494 and 124 ppt, respectively [46]. The pattern of detection at these sites showed that the emissions were dominated by local industrial activities such as the manufacture of polystyrene insulation boards.

The oxidation products of the HFOs and HCFOs are, in general, similar to those occurring from degradation of the HFCs and HCFCs, and include carbonyl compounds that have the potential to yield trifluoracetic acid (TFA), through hydrolysis or via secondary photochemistry [47]. Models indicate that a direct replacement of HFC-134a with HFO-1234yf in refrigeration applications will increase the associated global TFA burden from an annual 65 to 2220 tonnes formed from an equivalent emission of HFO-1234yf (based on emissions in 2015) [48]. However, given the low toxicity of TFA (see below), the increase in global environmental concentrations is not expected to significantly impact environmental or human health.

A common intermediate degradation product, trifluoroacetaldehyde (CF_3_CHO), can also interact with and promote the growth of secondary organic aerosols (SOA) [49]. However, this process is strongly dependent on season and the atmospheric conditions. The dominant removal pathway for CF_3_CHO is photolysis (lifetime around one day) [50]. The formation of SOA can also be affected by TFA, which has recently been shown to enhance the formation of dimethylamine-sulphuric acid particles (up to two orders of magnitude under certain atmospheric conditions) [51, 52]. This process can occur in winter with low sulphuric acid concentrations in the atmospheric boundary layer. With increasingly effective regulations on emissions of sulphur-containing pollutants, the effect of TFA on new particle formation may become increasingly important in some areas near the emission of precursors.

### Trifluoroacetic acid continues to be detected in the environment but at levels well below thresholds of toxicity

TFA has natural geochemical sources [53], is widely used in industry and research laboratories, and is a by-product of the synthesis and degradation of fluorinated and perfluorinated compounds (PFCs). Phototransformation on the surface of montmorillonite clay has been identified as a potential mechanism for the breakdown of longer chain PFCs (e.g. perfluorooctane sulfonamide (FOSA)) to shorter chain PFCs including TFA [54]. There are many other potential anthropogenic sources of TFA. The C-CF_3_ moiety, which is likely to be degraded to TFA as a terminal and very recalcitrant residue in the environment, is widely used in the synthesis of pharmaceuticals and pesticides. For example, in a compilation of the molecular structures of  > 1200 compounds used or proposed for use as pesticides, 228 contained a C–CF_3_ moiety [55]. Upon degradation or metabolism, some could produce TFA. Thus, the sources of TFA found in surface and marine waters are likely to be extremely diverse and uncertain. Because of lack of data on the other sources, it is not possible to quantify the proportion of anthropogenic sources resulting from substances falling under the purview of the Montreal Protocol and its Amendments. However, the presence of TFA in precipitation is most likely due to degradation of gaseous refrigerants and blowing agents, which do fall under the purview of the Montreal Protocol.

Further reports of the detection of TFA in surface waters have been published recently [48, 56, 57]. TFA has also now been detected in indoor dust in China. Concentrations were generally greater than those of the longer chain perfluorinated compounds, ranging from 100 to *ca* 500 µg kg^−1^, with the largest values from samples taken in the north-east and south-west of China [58]. The presence in indoor dust suggests a possible pathway of exposure for humans but exposure was not assessed, and a risk assessment was not conducted. TFA has also been detected in beer, tea, and other herbal infusions [59]. Median concentrations in beer were 6.1 µg L^−1^ and, in tea and other infusions, 2.4 µg L^−1^. Given the low toxicity in mammals [53], these amounts present a *de minimis* risk to humans. Uptake and translocation of TFA by plants from soil has been demonstrated [60], but whether the TFA in beer and infusions was formed from pesticides used in fields or from breakdown of replacements for ODS in the atmosphere is uncertain.

No further studies on the toxicity of TFA to organisms have been reported in the recent literature. Previous studies have shown that TFA is not highly toxic to aquatic organisms, although some plants (and algae) are more sensitive than animals. However, there are few tests on the toxicity of TFA to marine species. The only saltwater species tested were two unicellular algae [53]. No saltwater species of multicellular plants have been studied thus far. The paucity of data on sensitivity of marine plants to TFA is a potential source of uncertainty in the assessment of risks, especially as marine waters and endorheic lakes (lakes without an outflow) are the terminal basins for TFA, regardless of source.

## Climate benefits of the Montreal protocol

In addition to protecting the biosphere from harmful increases in UV radiation, the Montreal Protocol is helping to reduce global warming as many ODSs are also potent GHGs [61]. This section discusses a further beneficial effect of the Montreal Protocol that has recently been reported: by preventing excessive increases in surface UV-B radiation that would be harmful to terrestrial ecosystems, the Montreal Protocol is safeguarding the capacity of plants to sequester carbon dioxide (CO_2_) through photosynthesis and is thereby reducing increases in atmospheric CO_2_ concentrations that would lead to additional global warming.

### The Montreal Protocol is helping to curtail global warming by preventing excessive increases in surface UV-B radiation that would be harmful to plants

According to a recent modelling study by Young et al. [62], biologically effective UV-B (280–315 nm) radiation [63] would have increased by about 400% over the twenty-first century if the production of ODSs had not been controlled by the Montreal Protocol. The ensuing harmful effects on plant growth were estimated to result in 325–690 billion tonnes less carbon held in plants by the end of this century. This reduction in carbon sequestration would have led to an additional 115–235 parts per million of CO_2_ in the atmosphere, causing an additional rise of global-mean surface temperature of 0.5–1.0 °C. However, these estimates have large uncertainties and should be viewed with caution because the “generalised plant damage action spectrum” [63] used in the calculations does not account for the variety of plant responses across species and ecosystems. Furthermore, experiments (summarised by Ballaré et al. [64]) have not yet established whether the assumed sensitivity of plants to increases in UV-B radiation (i.e. a 3% reduction in biomass for every 10% increase in UV-B radiation for the “reference” scenario considered by Young et al. [62]) can be extrapolated to the enormous increases in UV-B radiation simulated in this study. For example, Young et al. [62] did not consider that plants have protective mechanisms against high amounts of UV radiation by synthesising UV-absorbing compounds [65–67], nor that they can develop adaptive morphological features (i.e. changes in their structure or form) [68]. Such adaptations would mitigate the net CO_2_ flux into the atmosphere. Conversely, enhanced photodegradation of organic matter under elevated UV radiation would release additional CO_2_ into the atmosphere [69].

## Interactive effects of UV radiation and extreme climate events on terrestrial ecosystems

Extreme climate events (ECEs^5^) are increasing in frequency and severity with climate change and are projected to become even more prevalent in the future as the climate continues to change [27]. Notable recent examples of ECEs include catastrophic floods, record-breaking snowstorms and droughts, heat waves, more frequent intense cyclones/hurricanes, rapid snow/ice-melt, and devastating wildfires [71–76]. All of these ECEs can have widespread, severe and long-term impacts on ecosystem stability and biodiversity [e.g. 77–80] and provide little opportunity for adaptation or mitigation measures [27]. Moreover, ECEs are superimposed upon ongoing trends of increasing global temperatures and atmospheric CO_2_ concentrations, as well as changes in other environmental factors [81], which have the potential to adversely affect many organisms.

### Extreme climate events modify the exposure of terrestrial ecosystems to solar UV radiation

**Fig. 3:**
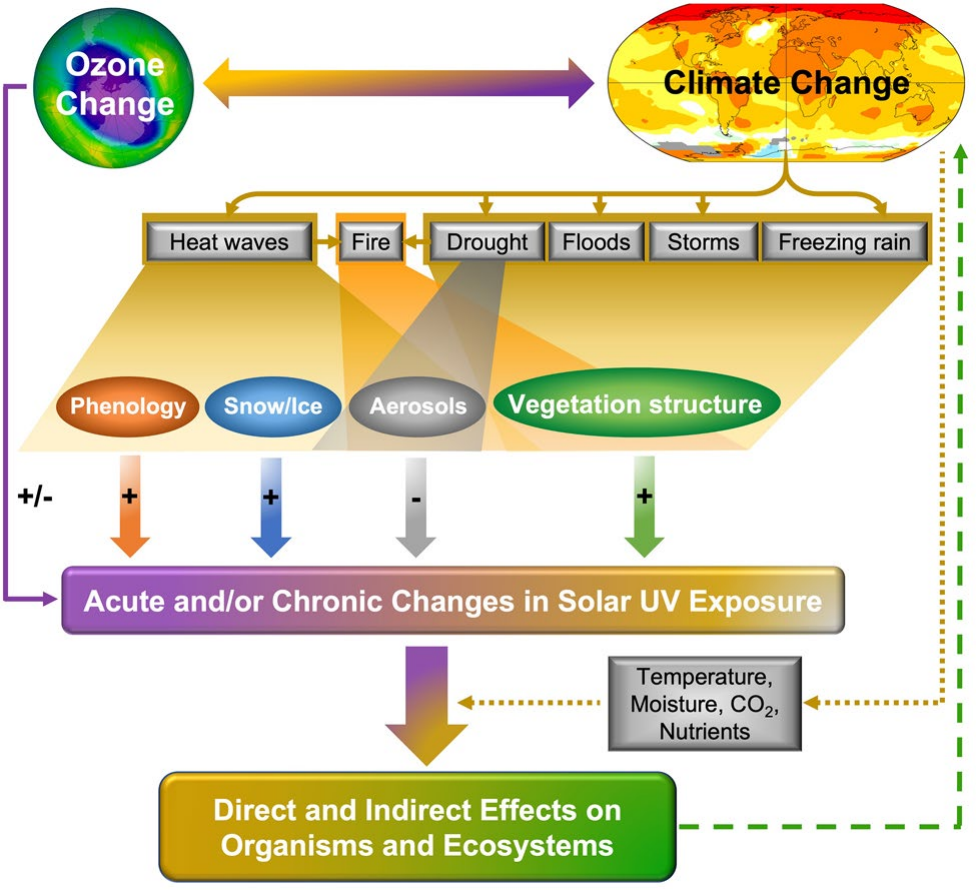
Pathways by which extreme climate events driven by changes in stratospheric ozone and climate can modify exposures and responses of terrestrial organisms and ecosystems to solar UV radiation (solid lines). Dotted line shows modulating effects of climate change factors on response to UV radiation, while dashed line indicates feedback effects of the biosphere on the climate system. Increases in exposure to UV radiation are shown as plus signs (+), and decreases as negative signs (−)

The changes in exposure of terrestrial organisms and ecosystems to solar UV radiation caused by ECEs include changes in atmospheric conditions (e.g. stratospheric ozone, aerosols and cloud cover), modification of land cover (e.g. snow, ice and vegetation), and alteration in the timing of development in organisms (i.e. phenology) (Fig. 3). The changes in exposure to UV radiation associated with ECEs can occur over short or long time periods, manifesting in acute or chronic ecosystem effects, respectively. Within the context of the Montreal Protocol, these changes in solar UV radiation together with other climate variables (e.g. temperature, moisture availability) can have detrimental effects on biodiversity, GHG emissions [1, 82, 83], carbon storage [81], ecological resilience, productivity of forests and crops, and lead to species replacement and potential deterioration of ecosystem functioning [84].

Many organisms use temperature as a cue to control the seasonal timing of their life stages, growth and development through the year (i.e. phenology), and ECEs that change temperature over a sustained period (heat or cold waves), have high potential to disrupt phenology [85, 86]. Given the seasonal variation in solar UV radiation, shifts in phenology can alter exposures to UV radiation and create new combinations of UV radiation together with other abiotic and biotic conditions to which species may not be adapted. Combinations of excessive UV radiation and other potential stresses can have detrimental effects on plant growth and survival, even though each individual stressor may only have a small effect [87].

### The effect of ECEs on terrestrial ecosystems in Polar Regions

The annual Antarctic stratospheric ozone hole and periodic severe Arctic ozone depletion increase solar UV radiation at ground level, although these events generally occur in early spring when many plant species are dormant and are often under snow cover [15]. However, when record-breaking increases in UV radiation and duration of the Antarctic ozone hole occur (Sect. 1), plants and animals may be negatively affected. Those organisms that live above the snow, or where snow cover has been lost due to persistent warm temperatures, would be uncovered, exposing them to acute high levels of UV radiation. For example, the return of many migrating animals to breed in Antarctica would have coincided with the persistent ozone hole in 2020, with the risk of exposure to an unusually high UV index in Antarctica from mid-November to late December.

Generally, extreme high temperatures lead to less snowfall and/or accelerated snow melt in cold regions, bringing forward the resulting flush of moisture and exposing ground flora and fauna to solar UV radiation earlier in the spring. However, UV radiation received by organisms above the snowpack may in some cases be reduced due to decreases in its reflection when the snowpack melts [88]. Reduced snow cover also permits more heat to be absorbed by the ground or low-lying vegetation in early spring, exacerbating the effects of warmer temperatures, melting permafrost and potentially leading to drier soils in the summer [86, 89]. Exposure to UV radiation can stimulate acclimation responses resulting in cross-protection that reduces the negative effects of water stress in many plants [90–92], albeit it can also produce cross-sensitivity in some species [93]. This serves as an example of how multiple climate changes and ECEs can combine with UV radiation to have synergistic effects on organisms that increase their severity.

In Polar Regions, variability and trends in the downward coupling from the stratosphere to the troposphere have contributed to changes in climate patterns on seasonal and inter-annual time scales [4, 7, 94, 95]. In Antarctica, interaction between stratospheric ozone change and the strength of the dynamical coupling from the stratosphere may be partially responsible at a regional scale for trends in surface temperature and precipitation, placing the stability of these fragile ecosystems at risk [96–101]. There is also concern that changes in atmospheric circulation patterns may lead to more sustained periods of cold winter and spring temperatures from mid-latitudes to Polar Regions [19], which could delay growth and reproduction until later in the year, thereby increasing the exposure to UV radiation of young leaves and buds.

### The impacts of ECEs on terrestrial ecosystems can occur over large geographical areas far beyond their immediate vicinity

Reduction in land cover and greater canopy opening occurs as a result of disturbances created by ECEs, as well as changes in land use and climate. Fires, floods, ice storms and hurricanes are among the most vivid examples of how ECEs transform the landscape by opening vegetation canopies to high solar radiation, while causing loss of productivity and biodiversity, and the release of GHGs [102, 103]. Following these ECEs, forest-floor species must adjust not only to acute or chronic increases in the solar radiation they receive but also to increased fluctuations in temperature and moisture. Some, but not all, plant species typical of forest-floor habitats respond quickly to the increased solar radiation, including UV-B radiation, by increasing their accumulation of protective pigments [104, 105]. However, recovery of ecosystems from these ECEs will depend on the scale and frequency of these events, with regeneration often failing to occur following the most severe events [106]. Recolonisation of disturbed sites will also depend on the types of species that thrive in the more open habitats created, their biodiversity value and traits that support ecosystem function. For example, the plants that colonise open habitats and tolerate high UV radiation may include more introduced and fewer specialised or endemic species [107, 108] which may, in turn, have negative consequences for biodiversity.

Increased abundance of aerosols resulting from wildfire smoke and the dust and plant volatile compounds released during droughts can decrease the amount of UV radiation and change the spectral composition of sunlight received by organisms at ground level [109]. Notably, changes in air quality resulting from fires and droughts can occur well beyond the location of these events [32, 110, 111]. These atmospheric changes are likely to modify photosynthesis and light-driven development in plants, as well as litter decomposition and emissions of GHGs from ecosystems [112] not directly impacted by the extreme events.

### The implementation of geoengineering interventions aimed at reducing global warming would likely generate ECEs and have wide-ranging impacts on terrestrial ecosystems

Solar Radiation Modification (SRM) has been proposed as one example of geoengineering to reduce the amount of solar radiation reaching the Earth’s surface through cloud brightening, cirrus cloud thinning, or injection of sulphates into the stratosphere [27]. Modelling studies indicate that, in addition to changes in climate, SRM could cause ozone depletion (mainly in Polar Regions) and ozone enhancements (mainly in the tropics and mid-latitudes) [113–115]. While SRM could offset some of the rapidly warming climate conditions, this intervention could lead to a number of disruptions in natural and agricultural ecosystems that could compromise food production and essential ecosystem services [116]. Substantial increases or decreases in UV radiation at the Earth’s surface would have further significant consequences for humans, animals, crop production and quality, and ecosystem sustainability [84]. At the present time, there are large uncertainties around potential risks and unintended consequences of SRM and other climate interventions.

## Biogeochemical cycles

Most research into the role of solar UV radiation on biogeochemical cycles has focused on the degradation of organic matter and contaminants on land and in fresh and marine waters. In both aquatic and terrestrial environments, solar UV radiation affects organic compounds in similar ways. In some cases, solar radiation drives the complete photodegradation of organic matter to CO_2_ and other inorganic compounds, referred to as photomineralisation. In addition to the effects of photodegradation on the release of CO_2_, solar UV radiation may also have an impact on the carbon cycle by affecting photosynthetic organisms that are major sinks for CO_2_ (Sect. 3). In other cases, partial photodegradation breaks down the original organic matter to produce a pool of organic and inorganic compounds, which may be more easily degraded by microorganisms—a process called photofacilitation. Although photodegradation plays a major role in removing toxic contaminants from the environment, partial photodegradation may produce intermediate products that are more toxic and/or persistent than the original compounds (Sect. 6).

### UV radiation contributes to decomposition in many terrestrial ecosystems by breaking down or changing certain compounds in plant litter

The role of photodegradation in litter decomposition is well established [84, 112]. There is now more evidence that photodegradation plays a key role in moist and temperate systems, such as in temperate forests [117], as well as in dryland systems [118, 119]. How much plant litter gets exposed to solar radiation, including the UV component, varies among ecosystems. In dryland ecosystems, litter is left on the ground in the dry season but burial can reduce exposure [119], while in temperate forests litter becomes exposed when gaps form in the canopy [117]. Fire also reduces vegetation cover and temporarily increases exposure to solar radiation [120].

Exposure to solar radiation, including the UV-B component, can change the chemical composition of litter even when there is no mass loss. For example, no effect of solar radiation was found on mass or total lignin content in temperate forest leaf litter. However, lignin oxidation was observed, suggesting possible photofacilitation in the longer term [121]. Furthermore, it has been demonstrated that exposure to solar radiation of terrestrial leaf litter in a temporary dry stream increased cellulose and lignin content [122], while in other instances UV radiation results in a net loss of cellulose and lignin [121].

### UV radiation affects nutrient cycling in both terrestrial and aquatic systems

Solar UV radiation affects nutrient cycling, as shown in a meta-analysis of field studies investigating the role of UV radiation on litter mineralisation [123]. Under reduced UV radiation, nutrient mineralisation was slow and poorly correlated with loss of litter mass, while under increased UV radiation, phosphorous and nitrogen mineralisation was fast and correlated with carbon mineralisation. This analysis suggests that microbial processes dominate nutrient cycling under decreased levels of UV radiation, while abiotic processes become more important as the intensity of UV radiation increases.

Mineral nitrogen and phosphorous are also produced in aquatic ecosystems during photodegradation of dissolved organic material (DOM), with potential effects on nutrient-limited ecosystems [124–126]. Work assessed in our earlier Quadrennial Assessments showed that UV radiation is involved in the photomineralisation of DOM to ammonium, a process called “photoammonification” [112, 127, 128]. New research shows that photoammonification changes in response to seasonal and climatic factors, explaining conflicting results from the literature on this process [129]. During the past year, there has been an increasing interest in understanding the role of solar UV radiation in triggering the release of phosphate. Not only DOM [130], but also suspended sediments [125] and river margins are subject to periodic flooding [131], releasing phosphate upon UV irradiation. This effect of UV radiation on release of organically bound nitrogen and phosphorus can have positive or negative consequences for the environment in terms of increased bioavailability or eutrophication of these forms of nutrients.

### Photodegradation of organic matter produces methane and a pool of biologically labile compounds

As global warming accelerates the melting of the cryosphere, further evidence has accumulated on the role of photofacilitation in Arctic surface waters [132–134]. It is well known that photodegradation facilitates the microbial degradation of organic matter in terrestrial and aquatic systems [112]. New insights into the mechanism of photofacilitation show that reactions induced by UV radiation are similar to those catalysed by the enzymes involved in the microbial degradation of DOM (e.g. decarboxylation) [134]. Thus, solar UV radiation not only contributes to the release of nutrients and bioavailable compounds, but also chemically alters DOM as microbial enzymes do.

In Arctic waters and boreal lakes experiencing browning, UV radiation, however, has a limited role in DOM mineralisation (e.g. release of CO_2_) compared to biological processes [135–137]. It is possible that previous research has overestimated the extent of photomineralisation in Arctic rivers because data have been modelled with a gas transfer velocity typical of lakes, an assumption that recent studies proved inaccurate [136]. The possible role of photofacilitation was not investigated in these studies.

**Fig. 4:**
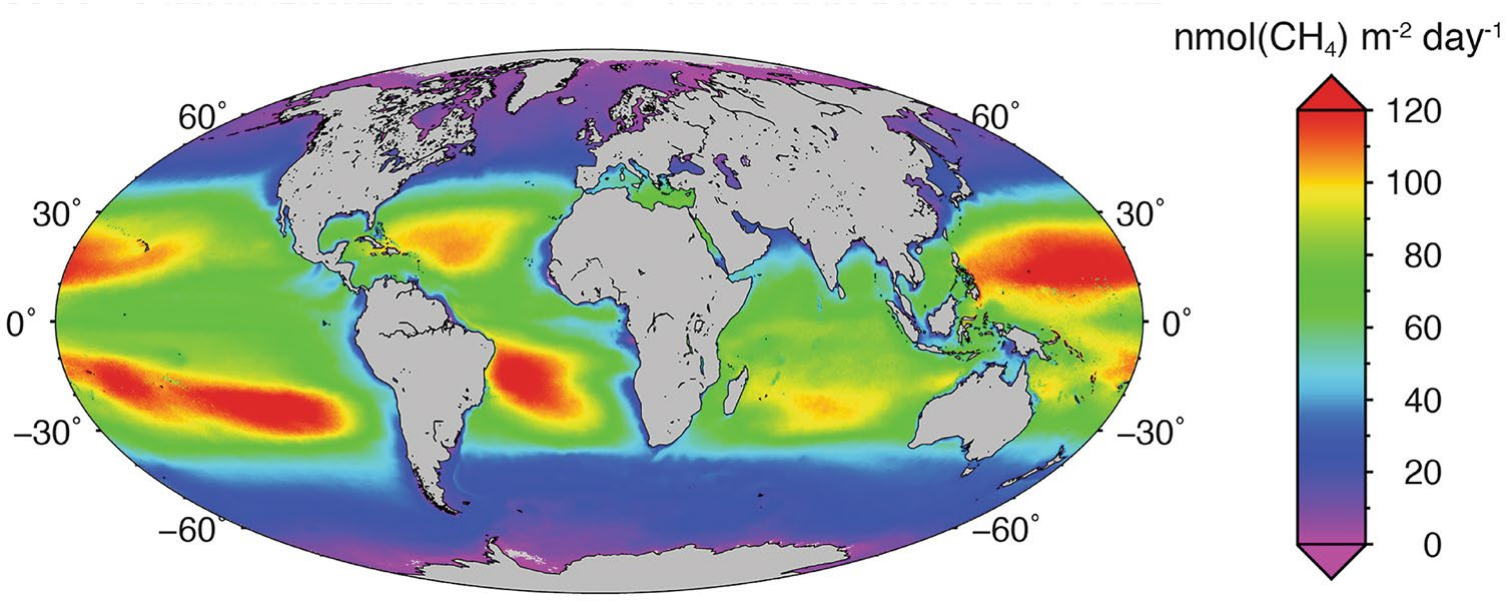
Depth-integrated methane photoproduction rate (top 150 m) calculated with a photochemical model based on remote sensing reflectance data (Figure by Rachele Ossola, adapted from Li et al. [138])

Photodegradation of DOM also produces CH_4_, potentially explaining why the ocean surface is supersaturated with this gas. In particular, photochemical reactions in subtropic ocean gyres are responsible for an annual production of 118 Gg of CH_4_ (35–71% of the total oceanic CH_4_ production), while contributions from coastal, sub-polar, and Polar Regions are minimal (Fig. 4) [138]. Photodegradation by UV radiation, with contributions from other wavelengths of solar radiation, together with microbial degradation therefore contribute to global warming through the release of CH_4_ and CO_2_.

### Photodegradation by UV radiation is a key driver of the breakdown of many contaminants

Photodegradation is a key mechanism by which organic contaminants in the environment are removed [139]. Understanding the photodegradation mechanism of a contaminant and its wavelength dependence is necessary for assessing its degradation pathway in the environment and, thus, potential health risks [140]. There has been an increasing interest in harnessing photodegradation to manage contaminants. For example, UV-B radiation induces the photolysis or breakdown of neonicotinoid insecticides, which are commonly applied to agricultural crops [141]; and wastewater treatments can maximise exposure to solar radiation in the field [142, 143] or through the use of novel photocatalysts [144].

New photodegradation models have been developed for organic contaminants. A review of apparent singlet oxygen quantum yields, a parameter needed to model indirect photodegradation, found a systematic overestimation of values in the literature that were measured with broadband radiation [145]. In parallel, the concept of equivalent monochromatic wavelength, the single wavelength that approximates the behaviour of a broadband radiation exposure, has been introduced to simplify the simulation of photodegradation rates of organic pollutants [146].

In some cases, photodegradation products are more persistent or toxic than the parent compound. For example, indirect photodegradation can transform some per- and polyfluoroalkyl substances (PFAS) into perfluorocarboxylic acids (PFCAs), which are considerably more persistent than PFAS [54, 147]. The potential toxicity of photodegradation products also requires consideration, even when the initial contaminant is not a cause of concern [148]. For instance, recent research indicates increased toxicity of decabromodiphenyl ether (BDE 209) solutions during photodegradation [149]. Decabromodiphenyl ether (BDE 209) is a widely used fire retardant.

The effect of solar UV radiation, and UV-B radiation in particular, on contaminant toxicity and breakdown depends on several factors, including the chemistry of the contaminant, the amount of UV-B and UV-A radiation (315–400 nm), and location in the environment. Thus, assessments of the net effects of solar UV radiation on contaminant toxicity often proceed on a case-by-case basis.

## Aquatic ecosystems

Climate change and implementation of the Montreal Protocol have modified the UV-B radiation received by aquatic organisms and ecosystems, affecting aquatic biodiversity and ecosystem services. In this section, we focus on the interactions of increased precipitation and runoff (often the consequence of extreme climate events), ocean warming, and wind intensity, and their influence on the exposure and responses of aquatic organisms and ecosystems to UV-B radiation. This adds to previously assessed research on variation of the exposure and effects of UV-B radiation with changes in ice cover and seasonality of the stratification of aquatic ecosystems into surface and deep layers [1]. New findings also include the modification of toxicity of oil pollutants by UV-B radiation.

### Extreme precipitation events are increasing inputs of dissolved organic matter into aquatic systems, shielding undesirable parasites, pathogens, and their vectors from disinfecting UV-B radiation

Exposure to UV radiation in aquatic ecosystems is reduced by increases in dissolved organic matter (DOM) that darken coastal and inland waters. This can be beneficial for water-borne parasites and their vectors by reducing exposure to disinfecting UV-B radiation and longer wavelengths of solar radiation [150]. Lakes with lower transparency tend to have larger and longer epidemics of both bacterial and fungal parasites of the widely distributed zooplankton grazer *Daphnia *[151]. Incubation of early-stage mosquito larvae in outdoor, temperature-controlled water baths demonstrated that solar UV radiation significantly decreases larval survival [152], whereas the addition of DOM significantly increased survival of mosquito larvae incubated in the presence of UV radiation. This suggests that DOM creates a refuge from damaging solar UV radiation [152]. Consequently, DOM may increase the success of mosquito breeding in shallow water bodies exposed to solar radiation. Extreme precipitation events may also increase the number and persistence of shallow pools and other potential breeding habitats for mosquitoes, such as uncleaned gutters, old buckets or boats left outside. Thus, climate change-related increases in heavy precipitation may act as a double-edged sword by increasing the refuge for UV-sensitive pathogens from disinfecting UV radiation and by creating more suitable habitats for mosquitoes, which are important vectors of disease in many regions of the world. In a related review about the prevalence of pathogens in shellfish aquaculture, the interactive effects of climate change and solar UV radiation on prevalence was identified as an important knowledge gap [153]. Collectively, these studies identify a critical role of UV-B radiation in regulating coastal and inland aquatic ecosystem services, including the threat of water-borne and vector-borne diseases to humans and wildlife, as well as to aquaculture and food security.

### Climate change is causing deepening of ocean surface-water circulation, decreasing the average exposure of organisms and materials to UV-B radiation in most regions

**Fig. 5:**
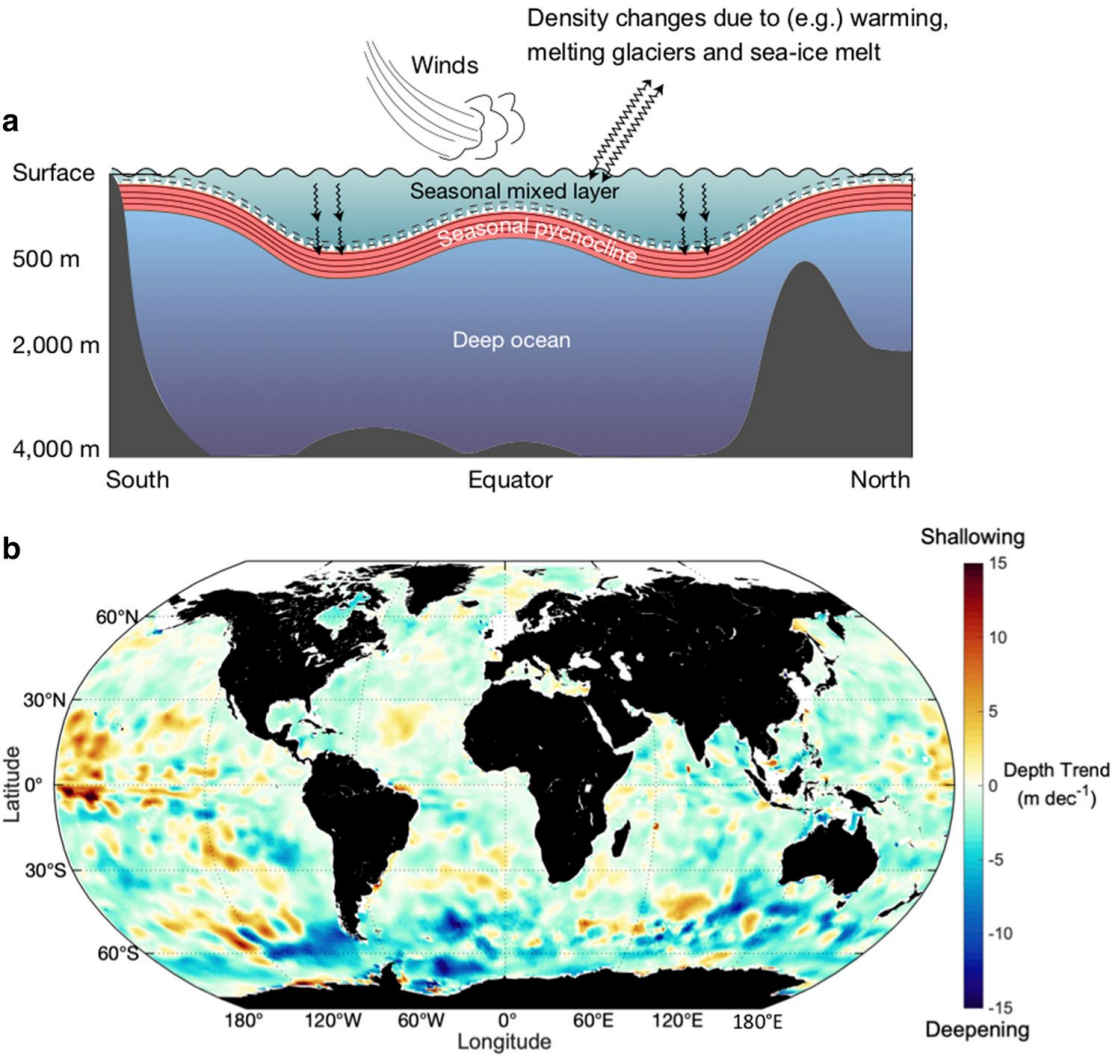
**a** Schematic illustration of the three-layer structure of the world ocean. The upper mixed layer is stirred by a range of turbulent processes driven by the wind. Warming, and glacier and sea ice melting have increased the density contrast (that is, stratification) between surface and deep waters that meet at the pycnocline (a layer of density transition between the upper and lower layers). Mixing incorporates water at the top of the pycnocline into the surface layer, deepening it, but also making the pycnocline sharper. **b** Global trends in ocean mixed layer depth during summer show localised regions of deepening at high southern latitudes (blue shading) and shallowing mostly at lower latitudes (orange shading). White indicates areas of zero or non-significant trends (modified from [154])

The water column in lakes and oceans is usually stratified in layers, with the layers having different densities due to different temperature and salinity. The surface mixed layer of the ocean, which is generally 20–100 m deep, is the warmest, most biologically active layer, and is the layer most vulnerable to UV-B radiation. In addition to the effect of water clarity on penetration of UV-B radiation into the layer, the depth to which the surface water circulates (the Mixed Layer Depth, MLD) determines the average exposure to UV-B radiation for suspended organisms and materials. The depth of the MLD is determined by the strength of the stratification (differences in density) between the MLD and the deep ocean and wind (Fig. 5a).

Observations of summer MLD from 1970 to 2018 in several global ocean databases showed an average deepening of 2.9% per decade, adding around 5–10 m per decade to the MLD [154]. The trend varies regionally, with less deepening in the North Atlantic (Fig. 5b). The deepening is contrary to earlier predictions that warming due to climate change should lead to shallower MLDs (e.g. [155]). The deepening implies a proportional lowering in average exposure to UV-B radiation of similar magnitude, and will reduce effects of UV-B radiation on organisms and photochemical processes (Sect. 5). Other effects of climate change over the observation period that deepen mixed layers, including intensifying surface winds, apparently outweighed the shallowing due to warming, although not everywhere [154]. For instance, shallowing is occurring near the Equator and in the Sub-Antarctic Southern Ocean (Fig. 5b). The Equatorial Pacific is an important region of high productivity [156]. If the shallowing trend continues in this region, aquatic ecosystems there may be at particular risk of increased effects of UV-B radiation, since incident UV-B radiation is projected to increase at low latitudes in the latter half of the twenty-first century due to decreasing cloud cover [1]. For more details on the interaction of UV, MLD and surface winds, see discussions in Williamson et al. [157] and Neale et al. [1].

### Exposure to UV radiation enhances the toxicity of oil pollutants, but reduces the toxicity of some other contaminants

Exposure to UV radiation amplifies the toxicity of certain contaminants in aquatic environments. These contaminants include polycyclic aromatic hydrocarbons (PAHs), which are formed from incomplete combustion and found in oil spills. While both UV-A and UV-B radiation amplify toxicity, UV-A radiation is responsible for most of the effects of solar UV radiation on PAH toxicity [158]. PAHs can accumulate in marine sediments, negatively affecting various organisms. For example, female fiddler crabs (*Uca longisignalis*) burrow in these marine sediments while incubating their eggs. After hatching, larval crabs have a free-swimming life stage in surface waters, where they can be exposed to UV radiation. Solar UV radiation resulted in increased mortality at the free-swimming larval stage after crab eggs were previously exposed to PAHs, indicating enhanced phototoxicity of bioaccumulated PAHs [159]. In addition, exposure to a combination of UV-B and UV-A radiation exacerbated the toxicity of heavy fuel oil to corals by 1.3-fold, including the early life stages such as the gametes, developing embryos, and planula larvae (the free-swimming dispersal stage) of the species *Acropora millepora *[160]. In contrast to PAHs, exposure to UV-B radiation can reduce the toxicity of other contaminants by catalysing their breakdown in the environment (Sect. 5).

## Natural and synthetic materials

Outdoor service lifetimes of both natural and man-made materials used in building or transportation sectors are determined primarily by their exposure to solar UV radiation during use. Any increase in the UV radiant flux in terrestrial solar radiation due to depletion of stratospheric ozone or higher average temperatures from climate change, will tend to decrease the average service life of materials. With most materials, such as wood and plastics, proven UV stabiliser technologies are available to counter a reduction in the useful lifetimes of materials due to increased solar UV radiation. However, their use adds significantly to the lifetime cost of materials used outdoors and has adverse environmental impacts, both from leaching of stabilisers and the persistence of stabilised plastics in the environment. The success of the Montreal Protocol and climate change treaties directly influence the environmental and economic cost of materials use.

An even more important consequence of increased solar UV radiation is the environmental cost of microplastics and nanoplastics generated by the photodegradation of materials^6^ that are routinely exposed outdoors. Potentially adverse impacts of these particles to the ecosystem, especially on the ocean environment, and on human consumers, are being studied to assess the magnitude of the risks they pose. To assess the overall impacts of increased exposure from solar UV radiation on materials use, it is necessary to consider the effectiveness of emerging UV stabiliser technologies, substitutes that may potentially replace conventional materials, the effect of changes in materials use due to sustainability considerations, the generation of particulate pollutants by their UV degradation, and the effectiveness of innovative textile fibre technologies that protect individuals from exposure to solar UV radiation. This section summarises significant developments in these areas.

### Recent detection of microplastics in human placenta suggests their presence in maternal systemic circulation

Two studies in clinical settings have detected microplastic contaminants in human placenta. In the first study, 12 polypropylene (PP) particles, 5–10 µm in size, were found in four placentae from vaginal deliveries [161]. In the second, larger microparticles > 50 µm of three plastics, polyethylene (PE), polypropylene (PP), and polystyrene (PS) were found in human placenta and meconium from caesarean delivery [162]. The location of microplastics on the foetal side of the placenta suggests that the placental barrier had been compromised by microplastics, even though transfer to the foetus has not been demonstrated. In rats, maternal inhalation of 20 µm PS nanoplastics has been shown to translocate into the foetus [163].

Photodegradation of plastics by solar UV radiation is primarily responsible for the generation of secondary micro- and nanoplastics [164], which are taken up by humans via inhalation, ingestion, and dermal contact [165, 166]. Airborne levels of microplastics (predominantly smaller than 100 µm in size) of 224 ± 70 particles/m^3^ and 101 ± 47 particles/m^3^ have been found in urban and rural air, respectively [167]. Inhaled microplastics and microfibres detected in lung tissue [166] may potentially access systemic circulation [168], resulting in adverse effects. While the role of environmental photodegradation of plastics exposed to solar UV radiation in generating micro- and nanoplastics is qualitatively established, quantitative relationships need to be developed between UV radiation and particle generation to better assess their global impacts.

### Novel, scalable and optically clear, solar UV-blocking wood composites show promise as a sustainable replacement for UV-screening glazing plastics in building applications

There has been a recent trend towards using novel, environmentally sustainable, wood-derived products that can potentially replace plastics in some building applications. Some of these novel composite materials also have better UV stability than the plastics and wood they replace. In an innovative technology, the lignin fraction of wood was partially removed and a synthetic [169] or biopolymer [170, 171] introduced into the residual cellulosic structure to obtain highly transparent wood composites (TWCs). Douglas fir wood/epoxy TWC show ~ 80% transmittance of visible light (for 2 mm thick samples) as well as UV-blocking capability with high absorption of UV radiation over the wavelength range of 200–400 nm [169]. Optically clear TWC materials that use natural polymers, such as chitosan or cellulose in place of the synthetic resin component, have also been synthesised [170]. With good thermal stability, up to 315 °C, TWCs can be designed to filter out UV-B radiation and have potential applications in smart building technology, especially in smart windows [172, 173]. However, even with the chromophoric lignin fraction partially removed, TWCs still undergo some photo-discolouration and degradation on extended exposure to solar UV radiation [174, 175]. This technical drawback, however, can be addressed with existing UV stabiliser technologies. For instance, blending 1.0 wt% of a conventional UV stabiliser into the epoxy resin used in one type of TWC, reduced the loss in transmittance of visible light on weathering. During 250 h of irradiation under UVA-340 fluorescent lamps, this resulted in only a 1.4% loss in transmittance of the stabilised TWC at *λ* = 550 nm compared to a 27.5% decrease in the untreated controls [174]. With improved UV stabilisation, this partially renewable and sustainable building material can become competitive with traditional building material replacing plastics in some products.

### Further studies demonstrate that nanoscale graphenes and reduced graphene oxides, increasingly used in polymer nanocomposites, also act as good UV stabilisers

Graphene and reduced graphene oxides (RGO) are especially popular nanoscale inorganic fillers used in composites due to their exceptional electrical, mechanical and anti-microbial characteristics [176, 177]. Advantages of graphene-based additives also include lack of polycyclic aromatic hydrocarbon contaminants and more sustainable production methods, compared to competing nanofillers such as carbon black [178]. In nanocomposites, graphene and RGO also impart UV stability to the polymer matrix at a low volume fraction [178]. For instance, a 5 wt% nanofiller mix of nano-cellulose/graphene (ratios of 2:1 to 16:1) in poly(vinyl alcohol) yielded a film that screened out 90% of solar UV radiation, with a 278% higher modulus (a measure of the stiffness of the material), and 59% lower water absorption, relative to the control [179]. RGO provided efficient UV shielding at a volume fraction of 3% in polyurethane coatings [180]. Similarly, a 2 µm thick cellulose film with a graphene oxide loading of 2% was visually transparent but still filtered out more than 54% of solar UV-B radiation [181]. A concern with their high-volume use in composites, however, is the potential release of nanoparticles due to weathering, especially under humid conditions [182, 183], during their use or disposal. The use of graphene-based nanocomposites will likely increase with products benefiting from the incidental UV stabilisation they also afford. However, their nano-release characteristics and eco-toxicological impacts during use, need to be better understood before their large-scale application.

### Synthetic microfibre fragments, generated by mechanical or degradative fragmentation by solar UV radiation, are abundant in ocean and freshwater sediments and biomass

Microfibres are the most prevalent type of anthropogenic particles in the ocean, often accounting for 80–90% of sampled microplastics [184, 185]. Polyester (PET) and nylon (PA) fibres, predominantly used in fabric, sink in seawater and are, therefore, under-represented in surface-water sampling. For instance, a global sampling of 916 microfibre samples collected from surface water of six ocean basins found only 8.2% of these to be synthetic plastics, while the majority (79.5%) was cellulosic, including both natural and man-made cellulose fibres such as rayon [185]. By contrast, microfibres sampled in industrial wastewater [186], in organisms [187, 188], algal biomass [189] or in the bottom sediment [190, 191], in both freshwater [187, 189–191] and marine environments [185, 188], show a high load of microfibres made of synthetic polymers.

Microfibres can be generated by fragmentation of textile fibres with or without exposure to solar UV radiation. Textile production processes [192–195] that involve abrasion [196] and electric dryers [197] as well as domestic laundering of clothes [198–200] mechanically generate microfibres in wastewater that reaches the ocean. However, the exposure of fabric to solar UV radiation also contributes to their degradation and fragmentation. Laboratory-accelerated weathering of PET and nylon (PA) fibres, the most used fibres, yield high levels of microfibres [201, 202]. For instance, textile fibres exposed to UV radiation in the laboratory, generally following the standard protocol (ASTM G155), resulted in fragmentation, with the average fibre dimension of PET and wool decreasing by 92% and 59%, respectively, after 56 days of exposure to UV radiation [201]. Nylons, however, did not show significant fragmentation over 56 days of exposure to UV radiation, but exhibited a 62% decrease in fibre length after 5 months of UV irradiation [201, 202]. While synthetic microfibres are clearly present in the ocean sediment and in biomass, the relative importance of photodegradation by solar UV radiation in generating them from textile fibres, as opposed to purely mechanical fragmentation, remains unclear.

## Human health

By protecting the stratospheric ozone layer, the Montreal Protocol has reduced the damaging health effects of excessive exposure to solar UV radiation. However, there are health benefits of moderate exposure to UV radiation, most notably vitamin D production. It is likely that by avoiding large increases in DNA-damaging UV-B radiation, the Montreal Protocol has allowed humans to safely tolerate time outdoors, thereby gaining the benefits of sun exposure. This may have reduced the risk or severity of a number of diseases, particularly those related to immune function, such as multiple sclerosis (MS) and COVID-19.

### Skin cancer continues to exert a considerable burden, although evidence of declining melanoma incidence in younger age groups in some populations continues to emerge

An analysis of Global Burden of Disease data found that in 2019, there were an estimated 4.0 million basal cell carcinomas (BCCs), 2.4 million cutaneous squamous cell carcinomas (SCCs), and 0.3 million malignant melanomas globally [203]. There were ~ 63,000 deaths due to melanoma and 56,000 due to SCC (and none due to BCC). Between 1990 and 2019 there was a global increase in the age-standardised incidence rate of all three cancer types. For melanoma, the largest increase was in East Asia, whereas for SCC and BCC (collectively called keratinocyte cancer (KC)), the greatest increase was in North America.

In Canada [204], Italy [205], and England [206] there is evidence that the incidence of melanoma is stabilising in younger age groups. The incidence in children aged 10–19 in the United States also decreased from 2000 to 2015 [207]. In Eastern Europe, studies from Lithuania (1991–2015) and Ukraine (2002–2013) reported increased incidence of melanoma in all age groups [208, 209], but there is evidence of a recent decline in incidence in Hungary [210].

An analysis of long-term trends in melanoma incidence in the United States, Denmark, and New Zealand reported average annual increases in incidence of 3–5% over the period 1943–2016. There has been a linear increase in the first two populations, but in New Zealand incidence has stabilised since the late 1990s [211], consistent with previous published reports. Thus, while positive trends in melanoma incidence in younger birth cohorts continue to emerge, higher exposure to UV radiation in older birth cohorts is most likely driving observed overall increases in incidence in many high-risk populations.

Keratinocyte cancers also show increased trends and a high burden in many countries. For example, in Iceland, age-standardised incidence rates of SCC tripled in males and increased by more than 40-fold in females between 1981 and 2017 [212]. In the United Kingdom, it has been estimated that one in five people will develop a KC in their lifetime [213], highlighting the importance of this disease even in a region with relatively low ambient UV radiation.

### Sunburn contributes to the health burden attributable to exposure to UV radiation, particularly in young men

Sunburn is common in light-skinned populations. In addition to being a risk factor for development of cutaneous melanoma and KC, inflammation from sunburn is itself a negative health outcome of exposure to UV radiation (primarily UV-B wavelengths). A recent cross-sectional study of the United States National Emergency Department Sample evaluated the burden of sunburns presenting to emergency departments (ED) in 950 hospitals [214]. Of 82,048 visits for sunburn, 21% were classified as severe sunburn. The average cost of an ED visit for sunburn was $1132. In the absence of the Montreal Protocol the incidence of sunburn is likely to have been considerably higher, due to the shorter time taken to burn, but the number of sunburns avoided has not yet been calculated.

### Eye diseases related to exposure to UV radiation continue to be a major cause of impaired vision

A meta-analysis that included 45 studies found an overall prevalence of cataract in adults aged ≥ 20 years of 17%. The prevalence of each of the two types related to exposure to UV radiation (nuclear and cortical) was ~ 8%. In people aged over 60 years, the prevalence of these cataract types was 31% and 25%, respectively [215], but there was considerable variability between regions.

Incidence data are available from two developed countries. In Finland, the incidence of cataract from 2000 to 2011 was 1% per year in people aged 30 years and over [216]. In the Singapore Malay Eye Study, ~ 14% of people aged ≥ 40 years developed a nuclear and ~ 14% a cortical cataract over a 6-year period [217].

### An increasingly recognised health effect of exposure to UV radiation is the suppression of pathogenic immune responses

UV radiation-induced immune suppression is hypothesised to explain the association between increased exposure to UV radiation and reduced disease activity in autoimmune diseases such as multiple sclerosis (MS) [218]. In a study of 946 people with relapsing remitting MS or Clinically Isolated Syndrome (CIS), a precursor of MS, living at higher latitudes or where satellite-derived estimates of ambient UV radiation are lower, was associated with worse MS severity scores. The risk of inflammatory lesions in the brains of MS patients (indicative of disease progression) increased by 8.3% for every 1° increase in latitude (risk ratio 1.08, 95% CI 1.01–1.16, *P* = 0.030) [219].

Another consequence of immune suppression may be reduced risk of eczema. In a study of 109 Australian infants, increased exposure to UV radiation was associated with reduced risk of eczema development, independent of vitamin D [220].

### Vitamin D deficiency continues to be a global concern, but one possible benefit of climate change is decreased deficiency in temperate climates

Evidence continues to emerge that the prevalence of vitamin D deficiency in various regions is high. A national study in Turkey between 2011 and 2016 found that 55% of people had a serum 25 hydroxy vitamin D (25(OH)D) concentration less than 50 nmol/L (classified as deficient by the United States Endocrine Society), and 27% were severely deficient [221].

In temperate climates, warming temperatures due to climate change may enhance the beneficial effects of exposure to UV radiation, most notably vitamin D production. In Germany, an analysis of 25(OH)D concentrations recorded at a large hospital clinic between 2014 and 2019 found that in the two ‘extreme’ (i.e. hotter and dryer than normal) summers, the prevalence of vitamin D deficiency was 10% lower than in other summers [222].

### Vitamin D, UV radiation, and other aspects of climate may play a role in the COVID-19 pandemic

There have been many publications investigating the link between the risk or severity of COVID-19 and UV radiation and/or vitamin D. There are, in principle, two mechanisms by which exposure to UV radiation could influence COVID-19: (1) Ambient UV radiation could inactivate the SARS-CoV-2 virus; (2) Vitamin D or other substances such as nitric oxide produced by exposing the skin to UV radiation could have beneficial effects on immunity and metabolism. Alternatively, UV radiation may be a proxy for other environmental factors (temperature, humidity) that cause people to spend time indoors in close proximity to others. In our last update assessment [1] we concluded that inactivation times from solar radiation (generally greater than 10 min) are too long to protect against outdoors transmission of SARS-CoV-2. Furthermore, most transmissions occur indoors where there is essentially no exposure to solar UV radiation. Regarding mechanism (2) or the role of other environmental factors, it is difficult to disentangle environmental effects from public health measures including social distancing, lockdowns, mandates to wear masks, and vaccination, which have varied markedly between countries and over time. Thus, the findings of the studies below should be interpreted cautiously.

There is some evidence that higher amounts of UV radiation are associated with reduced COVID-19 incidence. In an ecological study of over 19 million COVID-19 cases in 2,669 United States counties over 9 months, there was an almost linear inverse association between the SARS-CoV-2 reproduction number (*R*_*t*_) and ambient UV radiation, but only when UV radiation exceeded 100 kJm^−2^ per day [223]. However, UV radiation accounted for only 4.4% of the variability in *R*_*t*,_ compared with 3.7% for temperature and 9.4% for specific humidity. In another study, using global weather and infection data up to April 2020, ambient UV radiation, temperature, humidity, and the proportion of elderly in the population collectively explained 17% of the variability in the growth rate of SARS-CoV-2 positivity [224]. Of the environmental factors, UV radiation had the greatest effect. Similarly, an analysis of ~ 1.1 million COVID-19 cases in the early stages of the pandemic (up to mid-April 2020) from 3,235 geospatial units covering 173 countries across five continents found that higher surface UV radiation intensities lowered SARS-CoV-2 positivity growth rates [225]. A 10.7 kJ m^−2^ h^−1^ increase in ambient UV radiation increased the time taken for a doubling in the number of people who were virus positive from 5.2 to 5.7 days. Finally, in an analysis of data from 5 countries with different climate conditions and constant social controls of SARS-CoV-2 over the analysis period, low UV radiation was associated with higher viral spread [226].

COVID-19 mortality may also be affected by UV radiation. An analysis of mortality rates across areas of the United States, England, and Italy found ~ 30% reduction in the mortality rate ratio for every 100 kJ m^−2^ increase in mean daily UV-A radiation [227]. Similarly, in an analysis of data from 152 countries, a one unit increase in the UV index was associated with 1.2% lower growth rate in the cumulative number of deaths [228].

Induction of vitamin D may be one way in which exposure to UV radiation influences COVID-19. A meta-analysis found that people who were SARS-CoV-2 positive had lower concentrations of serum 25(OH)D than those who tested negative (mean difference (MD) 10 nmol/L) [229]. 25(OH)D was also markedly lower in people with severe *vs* mild disease (MD 17 nmol/L) and in people who died rather than being discharged from hospital (MD 20 nmol/L). However, there was high heterogeneity across studies. The measurement of 25(OH)D was not always concurrent with infection, and causality cannot be inferred from these studies.

Experimental studies investigating the effect of vitamin D supplementation on COVID-19 can help to determine if links with 25(OH)D concentration are causal. There have been several trials investigating the effect of vitamin D supplementation on outcomes in hospitalised patients. Two of these found that vitamin D was beneficial, but they were small and methodological issues call their findings into question [230, 231]. A gold-standard double-blind placebo-controlled RCT of 200,000 IU of vitamin D_3_ in 240 patients hospitalised with moderate to severe COVID-19 disease did not find effects on length of stay or in-hospital mortality [232].

Even if links with UV radiation are causal, the strong correlations between UV-B and UV-A radiation, temperature, and other environmental parameters make it challenging to determine the environmental factor responsible. Nevertheless, although less important than personal or geopolitical variables, climatic factors that are influenced directly or indirectly by the controls under the Montreal Protocol may have a small effect on the spread of SARS-CoV-2.

By protecting the stratospheric ozone layer, the Montreal Protocol has reduced the damaging health effects of excessive exposure to solar UV radiation. However, there are health benefits of moderate exposure to UV radiation, most notably vitamin D production. It is likely that by avoiding large increases in DNA-damaging UV-B radiation, the Montreal Protocol has allowed humans to safely tolerate time outdoors, thereby gaining the benefits of sun exposure. This may have reduced the risk or severity of a number of diseases, particularly those related to immune function, such as multiple sclerosis (MS) and COVID-19.

## Data Availability

All data generated or analysed are included in this article.

## Footnotes

1 The Arctic Oscillation (AO) or Northern Annular Mode (NAM) is analogous to the Southern Annular Mode (SAM) and characterises the pattern of winds circulating around the Arctic. When the AO is in its positive phase, a ring of strong winds circulating the North Pole acts to confine colder air in the Polar Region.

2 Shared socio-economic pathway (SSP) scenarios describe a range of plausible trends in the evolution of society over the twenty-first century and were adopted by the Intergovernmental Panel on Climate Change (IPCC) for its sixth Assessment Report. The pathways are used for climate modelling and research, as different socio-economic developments and political environments will lead to different GHG emissions and concentrations. They describe five climate futures, which differ in the amount of greenhouse gases that are emitted in years to come. The five SSPs (SSP1-1.9, SSP1-2.6, SSP2-4.5, SSP3-7.0, and SSP5-8.5) are named after a possible range of radiative forcing values in the year 2100 relative to pre-industrial values (1.9, 2.6, 4.5, 7.0, and 8.5 W m^−2^, respectively), and have some equivalence to the “Representative Concentration Pathways” or RCPs used in the fifth Assessment Report.

3 The global number of deaths from air pollution (particulate matter and tropospheric ozone) has been estimated to between 1.7 and 4.3 million per year [32]. In comparison, the number of deaths from skin cancer is about 120,000 per year (Sect. 8).

4 HFCs, hydrofluorocarbons; HCFCs, hydrochlorofluorocarbons; HFEs, hydrofluoroethers; HFOs, hydrofluoroolefins.

5 An extreme climate event has been defined as “an episode or occurrence in which a statistically rare or unusual climatic period alters ecosystem structure and ⁄or function well outside the bounds of what is considered typical or normal variability” [70]; or similarly, according to the IPCC, “if the value of a variable exceeds (or lies below) a threshold” [27]. Compound extreme events are the “combination of multiple drivers and/or hazards that contribute to societal or environmental risk.” An example of a compound extreme event would be fire weather conditions which are the combination of hot, dry, and windy conditions [27].

6 These secondary microplastics generally refer to fragments of plastics smaller than 5 mm in size, usually derived from photo-degradative fragmentation of plastics exposed to solar radiation. Secondary nanoplastics are similarly derived fragments that are smaller than 1 micron in size.
